# Top‐down and bottom‐up control of stress‐coping

**DOI:** 10.1111/jne.12675

**Published:** 2019-02-01

**Authors:** Edo R. de Kloet, Sybren F. de Kloet, Carien S. de Kloet, Annette D. de Kloet

**Affiliations:** ^1^ Division of Endocrinology Department of Medicine Leiden University Medical Center Leiden The Netherlands; ^2^ Department of Integrative Neurophysiology Center for Neurogenomics and Cognitive Research VU‐University of Amsterdam Amsterdam The Netherlands; ^3^ Foundation Center ’45 Arq Psychotrauma Expert Group Leiden The Netherlands; ^4^ Department of Physiology and Functional Genomics University of Florida Gainesville Florida

**Keywords:** adipose tissue, brain, cognitive flexibility, glucocorticoid receptors, limbic‐prefrontocortical circuitry, mineralocorticoid receptors, PTSD

## Abstract

In this 30th anniversary issue review, we focus on the glucocorticoid modulation of limbic‐prefrontocortical circuitry during stress‐coping. This action of the stress hormone is mediated by mineralocorticoid receptors (MRs) and glucocorticoid receptors (GRs) that are co‐expressed abundantly in these higher brain regions. Via both receptor types, the glucocorticoids demonstrate, in various contexts, rapid nongenomic and slower genomic actions that coordinate consecutive stages of information processing. MR‐mediated action optimises stress‐coping, whereas, in a complementary fashion, the memory storage of the selected coping strategy is promoted via GR. We highlight the involvement of adipose tissue in the allocation of energy resources to central regulation of stress reactions, point to still poorly understood neuronal ensembles in the prefrontal cortex that underlie cognitive flexibility critical for effective coping, and evaluate the role of cortisol as a pleiotropic regulator in vulnerability to, and treatment of, trauma‐related psychiatric disorders.

## 1 INTRODUCTION

As the late Seymour Levine once stated: “Stress is a composite, multidimensional construct, in which three components interact: (i) the input, when the stressor is perceived and appraised, (ii) the central processing of stressful information and (iii) the output or stress response. The three components interact via complex self‐regulating feedforward and feedback loops with the goal to restore homeostasis through behavioural and physiological adaptations.”^1^ There are physical stressors such as blood loss, pain, infection and inflammation that cause a reflexive activation of behavioural, autonomous, neuroendocrine and immune responses. More important for the current discussion, however, are the psychological stressors, which have a strong anticipatory component and rely heavily on central processing of information.^2^

The concept of stress can lead to misunderstanding because it has a negative connotation. Yet, the state of stress, namely the condition generated by the experience of the stressor and the stress response together, is essential for survival and is “the spice of life.”^3^ A healthy organism actually copes effectively with stress and learns from setbacks and adversity. In this case, the physiological defence reaction has met the environmental demand.^4^ On the other end of the spectrum is the failure to cope, an experience that can be worsened by fear, uncertainty, lack of social support and poor self‐esteem.^5^ This so‐called toxic stress enhances the vulnerability to mood and anxiety disorders.^6^

One challenge is therefore to understand how glucocorticoids, as master regulators of the stress response, can facilitate coping and adaptation. Another important quest is to identify the role of glucocorticoids in the mechanism underlying individual differences in coping with stress. The extent to which the stress hormone directs early‐life programming of stress and fear circuitry is a hot topic. Current evidence suggests a role of the pleiotropic glucocorticoids in precipitating vulnerability to stress‐related disorders, although the translation of this basic knowledge to clinical practice has been slow. All of these challenges ultimately lead to a fundamental question in stress research: How can the action of glucocorticoids change from protective to harmful? What is the cause and what are the consequences?

It is a sobering thought, however, that this question was equally important at the launch of *Journal of Neuroendocrinology* as it is today. Nevertheless, there is hope. Big data, as well as genome‐ and imaging technology, have revealed novel aspects of signalling cascades, circuit connectivity and synaptic plasticity that are at the root of the stress‐coping mechanism in higher brain regions. These regions are targets for the glucocorticoids that can coordinate and integrate the various stages of information processing, from perception and appraisal of a stressor to coping and behavioural adaptation. The naturally occurring glucocorticoids (corticosterone in rodents and cortisol/corticosterone in man) act via activation of two types of receptors: mineralocorticoid receptors (MRs) and glucocorticoid receptors (GRs), which were cloned around 1986,^7^ when the function of MR and GR was pharmacologically distinguished.^8^ Their properties and neuroanatomical localisation provided the rationale to study stress in the brain from gene to behaviour.^9^, ^10^, ^11^

Hence, in this 30th anniversary issue review, we use knowledge of MRs and GRs to sketch out how bottom‐up glucocorticoid action affects top‐down information processing in higher brain circuits during stress‐coping and adaptation. These actions exerted by the hormones require energy and, in this respect, we highlight the contribution of the fat‐brain axis^12^ (Box 1). We conclude with the possible role of glucocorticoids in vulnerability to post‐traumatic stress disorder (PTSD).^13^

Box 1 **Glucocorticoids, metabolism and stress**

1 To adequately cope with and adapt to stressors, it is essential that energy supply meets demand within the brain and other tissues that mediate this coping and adaptation. Depending on the circumstance (ie, whether the individual is actively or passively coping), the energetic requirement of the organism changes. Thus, glucocorticoids have profound and diverse actions at glucocorticoid receptors (GR) and at mineralocorticoid receptors (MR) in the brain and in peripheral tissues that alter metabolism and promote responses to a range of energetic demands. Centrally, glucocorticoids alter food intake and energy expenditure. Peripherally, glucocorticoids may act to mobilise, redistribute or even conserve energy. During times when energy demand is high, for example, glucocorticoids facilitate energy mobilisation by promoting gluconeogenesis in liver and proteolysis in muscle. In these instances, glucocorticoids also act in fat to stimulate lipolysis, thereby freeing fatty acids and glycerol into the circulation.^181^, ^182^

On the other hand, it is also widely accepted that some conditions induce glucocorticoids to facilitate the storage and/or redistribution of energy. Accordingly, within adipose tissue, glucocorticoids contribute to the formation of new fat cells (ie, adipogenesis) and to the growth of existing ones (ie, adipocyte hypertrophy).^12^, ^183^, ^184^, ^185^ Conceivably, this could be advantageous when the individual is anticipating the energetic cost of an upcoming stressor or is coping with a previously experienced threat. In line with this notion, enhanced long‐term actions of glucocorticoids within adipose tissue facilitate energy storage, as indicated by studies in rodents with altered glucocorticoid activity in adipose tissue,^182^, ^186^, ^187^, ^188^ and also by the profound metabolic effects of Cushing's disease.

So, collectively, glucocorticoids have a broad impact on metabolic tissues that allow an organism to meet the varying energetic demands of stress‐coping/adaptation. It is perhaps not surprising, therefore, that the secretion of glucocorticoids may, in part, be regulated by the peripheral metabolic target organs of the steroid. Metabolic factors influence hypothalamic‐pituitary‐adrenal (HPA) axis reactivity^189^ and it has been hypothesised that populations of GR in tissues involved in metabolism also regulate activity of the HPA axis.^190^ Moreover, using mice that lack GR in adipose tissue, our studies have revealed a key role for GR signalling originating in fat in the neural control of both stress and metabolism.^12^, ^182^ That is, mice with reduced adipocyte GR hypersecrete glucocorticoids following acute psychogenic stress and are resistant to diet‐induced obesity.^12^, ^182^ The broad implication is that glucocorticoid actions in adipose tissue influence central regulation of neuroendocrine stress responses and, as a consequence, may serve a functional role in stress coping/adaptation.

## 2 GLUCOCORTICOIDS

Glucocorticoids are pleiotropic signals for which it is difficult to discriminate between direct and indirect actions. The hormones regulate energy metabolism (Box 1), control immunity and inflammatory reactions to tissue damage, and have a profound action on brain function, behaviour and negative‐feedback action in the hypothalamic‐pituitary‐adrenal (HPA) axis. All chromosomes have a multitude of glucocorticoid‐responsive genes and many of these genes are themselves transcription factors. There is a strong sexual dimorphism in the actions of glucocorticoid.^14^ Most importantly, their action is diverse in every cell and tissue, which becomes manifest in a time‐ and context‐dependent manner.^15^ They bind to nuclear receptors involved in slow genomic actions, as well as membrane‐associated receptors regulating the release and action of transmitters, and also the functioning of ion channels.^16^

In rodents and man, glucocorticoids are secreted in hourly pulses. Their amplitude is largest at the start of the active period and stress responses are facilitated at the ascending arm of the pulse.^17^ Pulse amplitude and frequency may change during chronic stress, inflammatory disorders or major depressive disorder and, during senescence, the rhythm may become disorganised.^18^ Because pulsatile exposure to glucocorticoids is a determinant of target responsivity, such changes in ultradian rhythmicity may have profound consequences for resilience.^17^ Accordingly, glucocorticoid resistance is often the consequence of flattening of the ultradian rhythm, as occurs during major depressive disorder.^19^ Alternatively, the lower but more dynamic circadian pattern of circulating cortisol level observed in a subgroup of PTSD patients^20^, ^21^, ^22^ appears to be a vulnerability factor imposed by traumatic (early) life experience.^23^, ^24^

Glucocorticoids are bound in blood to corticosteroid binding globulin. In rodents and man, penetration into the brain is hampered for cortisol (and synthetic steroids), although not for corticosterone, by multidrug resistance P‐glycoprotein localised in the blood‐brain barrier. In humans, the ratio of corticosterone vs cortisol consequently rises from 1:10 in blood to 4:10 in the brain.^25^ The 11‐dehydro congeners of cortisol and corticosterone are inactive, although bioactivity can be regenerated intracellularly by 11β‐hydroxysteroid dehydrogenase type 1 (11HSD‐1). In most glucocorticoid‐responsive targets, this enzyme therefore ensures bio‐availability of glucocorticoids.^26^

The isoform 11HSD‐2 converts cortisol and corticosterone into their bio‐inactive congeners; for example, in kidney and colon epithelial cells that are engaged in Na^+^ retention. This inactivation of the glucocorticoids makes the MR *aldosterone selective*.^27^, ^28^ However, co‐localisation with 11HSD‐1 renders MRs *cortisol/corticosterone‐preferring*, and this is the case in the limbic brain, heart and adipose tissue. Despite comparable affinity for aldosterone, the limbic MR type prefers the natural glucocorticoids because, even at trough levels, their concentration exceeds that of aldosterone by 10‐ to 100‐fold. There is evidence that MR specificity depends on the intracellular oxido‐reductase balance. Also, the receptor shows a high affinity for a range of other steroids including progesterone and deoxycorticosterone. This promiscuity and near saturation of MR under all circumstances suggests that receptor activity rather than ligand concentration is the rate‐limiting step.^29^

Glucocorticoid receptors are expressed in every cell but bind cortisol and corticosterone with 10‐fold lower affinity than the MRs do. Thus, although MRs are already largely occupied with ligand at the diurnal trough, the GRs only become activated by rising glucocorticoid levels during stress and at the circadian peak.^8^ Depending on the hormone concentration, and still poorly understood cofactors, either MR‐MR and GR‐GR homodimers, in addition to MR‐GR heterodimers, are formed.^30^ Transactivation can become receptor‐specific by interaction with other transcription factors such as NeuroD^31^ or coregulators.^32^ Transrepression is privileged for GR interaction with transcription factors such as nuclear factor kappa B (NF‐κB).^32^

Mineralocorticoid receptor and GR knockouts are not viable but survive if the GR mutant carries a point mutation (A458T) that prevents homodimerisation, whereas transrepression by the monomer is preserved. These mice show impaired cognition, altered metabolism and compromised immune function, whereas corticotrophin‐releasing hormone (CRH) and pituitary corticotrophin release are not affected.^33^, ^34^

## 3 MR AND GR FUNCTION IN BRAIN

Within the brain, *aldosterone‐selective* MRs, characterised by 11HSD‐2 co‐expression, are restricted to the nucleus tractus solitarius (NTS).^35^, ^36^ In this discrete periventricular brain stem region, aldosterone‐selective MRs are involved in salt‐appetite and volume regulation.^37^ A very recent neuro‐anatomical tracing study^36^ demonstrated that a selection of NTS neurones project to the parabrachial/locus coeruleus nuclei, whereas other NTS neurones innervate the ventrolateral bed nucleus of the stria terminalis (BNST) and have some scarce projections to the ventral tegmental area (VTA), central amygdala and hypothalamus. These distinct targets are considered to modulate motivational arousal, and sensations of reward or disgust, and may explain cognitive functions, linked to the role of aldosterone in salt appetite.^38^ The NTS‐forebrain targets may be a substrate for anxiety and depression in patients suffering from hyperaldosteronaemia,^39^ and the behavioural effects of aldosterone observed in animal studies.^40^ Actually, components of the renin‐angiotensin‐aldosterone system, such as angiotensin‐II, also participate in coordination of the stress response (Box 2).

Box 2 **Mineralocorticoid receptors (MRs) and the renin‐angiotensin‐aldosterone system are pleiotropic**

1 In evolution, the renin‐angiotensin‐aldosterone system and the MRs regulate energy metabolism and electrolyte homeostasis in species as early as bony fish.^191^ MR is the primordial receptor for glucocorticoid receptors (GRs) and progesterone receptors (PRs) which may explain its promiscuous nature in binding with high affinity to aldosterone, deoxycorticosterone, cortisol, corticosterone and progesterone.^192^ Aldosterone‐selectivity emerges in terrestrial animals, as the end‐product of the renin‐angiotensin‐aldosterone system.^192^

These primordial pleiotropic functions of the renin‐angiotensin system are maintained in mammalian species. For example, angiotensin II regulates metabolism and is a key factor in obesity and associated inflammation.^193^, ^194^ Furthermore, the peptide activates a specific population of paraventricular nucleus neurones engaged in coordination of cardiovascular, behavioural, sympathetic and neuroendocrine responses to stress.^195^

Aldosterone‐selective MRs are expressed in discrete neuronal cell groups of the nucleus tractus solitarius (NTS) that innervate specific limbic‐prefrontocortical circuits involved in cognitive and emotional aspects of salt appetite.^38^ Abundant innervation by NTS axons occurs to parabrachial/locus coeruleus and of GABAergic neurones surrounding the ventrolateral bed nucleus of the stria terminalis, presenting a possible substrate for the depression and anxiety effects of hyperaldosteronism.^39^ Aldosterone secretion is triggered by angiotensin‐II to maintain salt homeostasis, but also in response to stress in hypertensive patients.^196^ Interestingly, in a subset of NTS neurones, GRs are expressed which mediate glucocorticoid feedback on hypothalamic‐pituitary‐adrenal axis regulation.^197^

In animal studies, aldosterone, as well as angiotensin‐II administration can evoke fear.^40^ Untreated patients suffering from primary aldosteronism have serious mental health problems: quality of life is compromised and frequency of depression and anxiety is increased.^198^ Antidepressants are known to induce MR synthesis,^199^ and attempts to link aldosterone‐MR biomarkers to treatment outcome in mental disorders are promising for hyperaldosteronism.^36^, ^200^ Alternatively, in a randomised, double‐blind, proof‐of‐concept study, the MR agonist fludrocortisone, rather than spironolactone, improved efficacy of antidepressants.^201^ Post‐mortem MR expression was found to be decreased in the limbic brain of depressed patients,^202^ whereas a common gain‐of‐function MR haplotype protected against depression.^203^ In conclusion, brain angiotensin receptors, as well as aldosterone‐selective and glucocorticoid‐preferring MRs, are promising but under‐investigated, candidates for therapeutic intervention in stress‐related mental disorders.

In limbic regions, not only notably the hippocampal pyramidal neurones, dentate gyrus, lateral septum and amygdala, but also in cerebellar and cortical regions, *glucocorticoid‐preferring* MRs are highly expressed.^41^, ^42^ Pharmacological and genetic manipulations have revealed that the *glucocorticoid‐preferring* genomic MR is an important determinant in the control of the sensitivity, responsivity and threshold of the stress response system.^43^, ^44^, ^45^ Nongenomic MR function is prominent in promoting the initial phase of the stress response where it facilitates autonomous functions important for attention and vigilance.^46^ Moreover, rapid MR activation is important for appraisal processes, behavioural reactivity and the selection of coping style. MR promotes the encoding of an experience for learning, at the same time also enhancing retrieval of previously acquired information.^47^, ^48^, ^49^, ^50^ With higher corticosterone concentrations, GRs become activated in stress circuitry, which facilitates behavioural adaptation and memory consolidation, amongst other effects.^47^, ^48^

At the cellular level, genomic MR activation maintains a high and stable excitatory tone in hippocampus and amygdala, enhances neurogenesis and reduces apoptosis observed in the dentate gyrus.^51^ In dorsal hippocampal neurones, membrane MR can rapidly activate miniature excitatory postsynaptic currents, which reflect the increased probability of glutamate release.^52^, ^53^, ^54^ MR stimulation facilitates the induction of long‐term potentiation (LTP).^55^ By contrast to the MR‐induced miniature excitatory postsynaptic currents in the dorsal hippocampus, miniature inhibitory postsynaptic currents are reduced in the ventral hippocampus.^56^ These processes in the dorsal and ventral hippocampus cooperate and result in an enhanced excitatory outflow. However, the dorsal hippocampus is more involved in cognitive processes in contrast to the function of the ventral part in regulation of emotion and affective state.^57^

Glucocorticoid receptors are expressed in all cells and occur in highest abundance in typical stress regulatory centres, such as the hypothalamic paraventricular nucleus, hippocampus, amygdala, ascending aminergic neurones and prefrontal cortex.^58^, ^59^, ^60^ Glucocorticoids activate via GR bio‐aminergic neurones. For example, in the VTA‐A10 circuit, the synthesis and release of dopamine is enhanced, which boosts reward processing, motivational arousal and reinforcement learning.^61^ Also, in the prefrontal cortex, GR has specific actions that depend on the severity and duration of the stressor.^62^ In addition, GRs mediate rapid nongenomic actions involving the release of endocannabinoids, which may block transmitter release and HPA axis activation trans‐synaptically.^63^, ^64^

In hippocampal CA1 pyramidal neurones, MR‐induced excitability is suppressed and normalised by a genomic GR‐mediated action and these opposing actions provide a U‐shaped dose‐response curve to corticosterone.^65^, ^66^ However, in the dentate gyrus, MR and GR activations act in the same direction and show a linear dose‐response relationship. Membrane MR‐ and genomic GR‐mediated actions can cooperate and promote metaplasticity of basolateral amygdala neurones^67^; this implies that excitability is rapidly increased via the membrane MR and subsequently maintained upon activation of the genomic GR. Such an action of glucocorticoids via both receptors was found to be further enhanced if combined with norepinephrine (NE) exposure.^68^ This synergising effect of glucocorticoids and NE may explain why emotional experiences are best remembered.^69^, ^70^

Ca^2+^ signalling in hippocampus and amygdala is another mechanism linking steroid action with brain function. Low levels of corticosterone activating the hippocampal MRs cause small L‐type Ca^2+^ currents, which increase in amplitude with rising corticosterone levels in a process requiring DNA binding of GR homodimers. Initially, during the first hours after stress, the rising Ca^2+^ influx contributes to increased frequency accommodation in hippocampus and amygdala and prevents the induction of LTP. These Ca^2+^‐dependent processes normalise stress‐induced excitability and help to protect encoding for memory processes from disruption by unrelated stressful information.^71^

**Figure 1:**
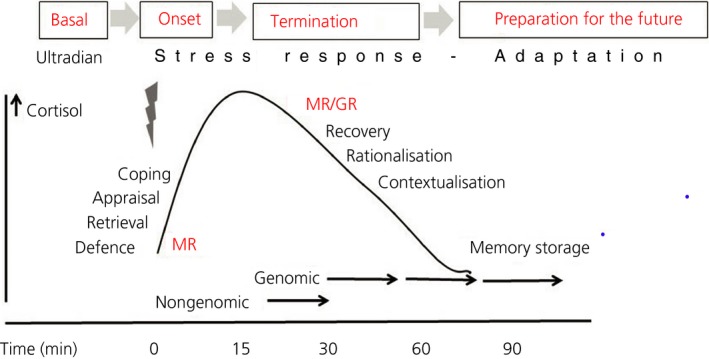
The trajectory of information processing proceeds from mineralocorticoid receptor (MR)‐dominated signaling cascades that activate the circuits underlying appraisal processes and selection of an appropriate coping style, when encoding the experience for learning, which are all geared to protect the “self.” Then, glucocorticoid receptor (GR)‐mediated actions are engaged with recovery by dampening cellular stress reactions and activating executive functions aimed towards rationalisation and contextualisation of the experience.^75^ At the same time, GR‐mediated actions promote behavioural adaptation and memory consolidation and, by doing so, prime brain circuits to be prepared for future threats in similar contexts. In the case of a recurrent event, such GR‐mediated imprints provide the substrate for retrieval by a MR‐mediated mechanism, and so on. Initially, MR homodimers are involved but, by increasing glucocorticoid concentrations progressively, MR‐GR heterodimers, GR‐GR homodimers and complexes of MR/GR monomers with other transcription factors may be formed. Adapted from de Kloet et al^10^

Processes under the control of MR promote the initial phase of the stress reaction.^72^ When the stress response proceeds, circulating glucocorticoid concentrations rise and the hormone starts, via GR, to prevent these initial defence reactions from overshooting and becoming damaging.^73^ At the same time, this later GR‐mediated action promotes memory consolidation of the experience. The different phases of the glucocorticoid stress response are shown in Figure 1.^11^, ^74^, ^75^ The findings have led to the formulation of the MR:GR balance hypothesis, which states that: “upon imbalance in MR:GR‐regulated limbic‐cortical signalling pathways, the initiation and/or management of the stress response is compromised. At a certain threshold, this may lead to a condition of HPA axis dysregulation and impaired behavioural adaptation, which can enhance susceptibility to stress‐related neurodegeneration and mental disorders.”^9^, ^19^, ^45^, ^75^, ^76^, ^77^, ^78^ Research on the molecular underpinnings of the coordinate MR‐ and GR‐mediated actions is reported elsewher,e^11^, ^79^ as well as in brief in the section on coping with stress below.

## 4 COPING WITH STRESS

The classic homeostatic mechanisms, which are essential for maintenance of electrolyte balance and body temperature, appeared to be insufficient to conceptualise stress‐related mental processes. Allostasis or adaptation to change, rather than recovery back to the original homeostatic state, better captures the processes driven by anticipatory components of psychological stressors. Allostasis describes an energy demanding process enabling the maintenance of a labile, or rather metastable, allostatic (new homeostatic) state. The energy expenditure required has been termed allostatic load.^80^, ^81^ According to Nuno Sousa, a “stressed brain” construct is hypothesised, whereby, upon chronic stress a “point of no return”^82^ is passed, implying that brain plasticity gets “stuck”^6^ and interventions are required to regain resilience. A key component in the theory is the role of glucocorticoids in providing sufficient energy to meet the demand required for allostasis during coping with stress.^83^, ^84^ Below, we specify the role of the MR in selection of a coping style and of GR in storage of the selected coping style in memory.

### 4.1 MR and selection of coping style

A striking example of the selection of coping style is revealed in tests that allow discrimination between hippocampal spatial learning and striatal stimulus‐response strategies in the location of an escape route. Naïve unstressed mice use a spatial strategy on a “hole board” to find the exit, allowing escape to the safe environment of the home cage. After a stressor, or injection of corticosterone, around half of the mice memorised the exit using stimulus‐response behaviour (or habit learning), which is linked to the dorsolateral striatum (DLS), whereas the other half maintained a hippocampal spatial strategy. The switch to stimulus‐response learning allowed the animals to maintain their performance, apparently by bypassing slower cognitive processing involving the hippocampus.^85^ Prior treatment with the anti‐mineralocorticoid RU28318 prevented the switch to habit learning, and again re‐introduced hippocampal function, although performance did not improve. Interestingly, female mice maintained the spatial strategy when exposed to stress.^86^

Schwabe et al^87^ discovered that the MR‐dependent switch also operates in humans.^87^ In these experiments, individuals were stressed and subsequently performed a cognitive task that involved both the hippocampus‐explicit and the DLS‐implicit memory systems when being monitored by event‐related functional magnetic resonance imaging. The cognitive task was based on probabilistic learning, the so‐called weather prediction task. In a sub‐set of individuals, stress caused a switch towards the DLS‐based implicit memory, which was prevented by anti‐mineralocorticoid treatment. MR blockade with spironolactone preserved hippocampal learning, although this was still significantly impaired. Using functional magnetic resonance imaging, it was again demonstrated, this time in humans, that the stress‐induced behavioural switch was paralleled by increased amygdala‐striatal connectivity at the expense of the amygdala‐hippocampus connection.^87^, ^88^, ^89^ Subsequently, carriers of the gain‐in‐function MR haplotype 2 gene variant, known to be associated with resilience and optimism,^90^, ^91^ readily switched to a preferred amygdala‐DLS connectivity, an observation that was supported by an electroencephalogram.^92^

Thus, MR activation facilitates a stress‐induced switch from hippocampal cognitive to striatal habit learning to optimise coping. The switch likely involves amygdala input to the striatal region, which, via a feedforward cascade of intrastriatal‐medial prefrontal cortex (mPFC) interactions,^93^ culminates in activation of the sensorimotor DLS. This stress‐induced MR‐dependent switch towards DLS‐based learning and memorising habitual behaviour is reminiscent of the outcome of exposure to a repeated uncontrollable stressor that also showed activation and hypertrophy of the DLS.^94^

### 4.2 GR and memory storage

Another example that links glucocorticoids with memory storage of the selected coping style is the performance of rodents in the “forced swim test” (FST).^95^ In this test, which was classically used to screen the efficacy of antidepressants, rodents are immersed for 15 minutes in a beaker filled with water from which escape is not possible.^96^, ^97^ Initially, the animal shows vigorous attempts to escape by swimming, struggling and climbing (active coping) but, after some time, an immobile floating posture is achieved (passive coping). Because there are no escape possibilities in this acute stressful situation, the animal's progressive transition from the use of more counterproductive active behavioural strategies to more energy‐conserving, passive ones is considered to be an example of a successful coping style.^98^, ^99^

In the original Porsolt FST, the retention of acquired immobility was scored 24 hours after training, in a 5‐minute retest. Here, adrenally intact animals immediately assume the immobile position, whereas antidepressants interfere with memory retention of the passive coping response and reduce immobility in the retest.^100^ Adrenalectomy (ADX) does not affect the rate of acquiring immobility but interferes with its memory retention. Glucocorticoid replacement of the ADX animal immediately after the initial forced swim normalises retention measured 24 hours later. Anti‐glucocorticoid treatment immediately after training in these glucocorticoid‐substituted ADX‐, as well as in adrenally intact animals, interfered with memory storage of acquired immobility. This effect of the anti‐glucocorticoid was noted after milligram amounts were given systemically or nanogram doses were adminstered locally into the hippocampal dentate gyrus.^99^, ^101^ The data demonstrate that glucocorticoids are important for consolidation and retention of acquired immobility. The consolidation mechanism was found to be localised in discrete neurones of the dentate gyrus. It involves convergence of stress‐induced NMDA and GR signalling pathways that could affect histone acetylation, chromatin remodelling and c‐Fos activation by demethylation its promotor, although only in a surprisingly discrete subset of dentate gyrus neurones.^102^, ^103^ FST‐induced transcriptome analysis revealed profound differences in specific genes, particularly those related to the NF‐κB pathway.^104^ This NF‐κB signalling cascade was previously found to be differentially expressed in the hippocampus of mice selected for high and low aggressiveness.^105^

Other studies revealed that the DLS is also involved. These studies are based on mouse lines that show extreme differences in transition of active to passive coping style. DBA mice show a slow transition and maintain active coping for a long time, whereas C57BL/6 mice readily acquire immobility. Using a c‐Fos marker and discrete lesions, it was established that the DBA mice relied on left‐hand DLS‐based learning of immobility, whereas the C57s used the dorsal hippocampus to acquire and retain immobility.^106^ DBA mice expressed more D2 receptors in the left DLS, and lesioning or blockade of the D2 receptors interfered with retention of the passive coping in the DBAs.^107^

The DLS controls habitual, stimulus‐driven, behaviour.^108^ In a modelling study, Fiore et al^109^ reached the conclusion that the involvement of the DLS was the result of a switch in prelimbic–dorsomedial striatum control involved in goal‐directed behaviour towards the infralimbic area that governs the stimulus‐response mode when the animal is unable to gain control over the situation.^109^, ^110^ Such a prelimbic‐infralimbic switch to acquisition of passive coping is assumed to enable the conservation of energy resources.

In sum, the animal's progressive transition from the use of more counterproductive active behavioural strategies to more energy‐conserving, passive ones is an example of successful adaptation to a stressful situation, which is promoted by GR activation. Therefore, transitions between active and passive coping strategies support adaptive behavioural decision‐making, and the behavioural flexibility to support these transitions is a welcome trait in fluctuating, natural environments.

### 4.3 Genetic selection of coping style

The outcome of attempts to deal with (in)escapable challenges relates to Henry's distinction of two evolutionary successful response modes in coping with psychosocial stress: the active fight‐flight and the passive conservation‐withdrawal mode.^111^ Koolhaas et al^4^, ^112^ and de Boer et al^4^, ^112^ have further elaborated the physiological and behavioural stress response patterns of these two extremes in coping style. In their studies, they used male mice genetically selected for highly aggressive short attack latency (SAL) vs long‐attack latency mice (LAL). A higher trait‐aggressiveness in these mice is associated with prolonged active coping bouts in the FST. By contrast, the LAL mice showed signs of withdrawal upon social defeat. The latter expression of a passive coping style triggered a much higher and prolonged release of corticosterone.^113^

The dominant SAL animals feature diminished cognitive flexibility, a rigid rather routine‐like behavioural control, a reduced impulse control, high sympathetic activity, high striatal DA activity and low HPA axis activity in response to stressors.^4^, ^112^ Such animals also have high limbic expression of MR and low 5‐HT activity, whereas MR antagonists can reverse some aspects of this phenotype.^114^ The active mice were qualified by Koolhaas et al^4^ and de Boer et al^112^ as *pro‐active* rather than active copers to reflect their tendency to take the initiative. On the other extreme, the passive animals, qualified as *reactive,* showed the mirror image: high HPA axis and parasympathetic activity but low sympathetic reactivity and low circulating testosterone levels. The “reactive” copers are much more sensitive to, and guided by, environmental influences.^112^

In their seminal studies, Henry and Stephens^111^ linked the amygdala to fight‐flight and the hippocampus to conservation withdrawal.^111^ Now, a few decades later, it appears that there is no brain region that does not respond during coping with stress. Rather coping constitutes a fluent process of complementary stages from processing of salient sensory information, to various executive functions.^89^, ^115^ Hence, a “social decision‐making network”^116^ overarching the salient and executive distinctions has been proposed that is ultimately responsible for the expression of various coping styles. This network originates from the mPFC, a locus for premonition, working memory, rationalisation and, of course, decision‐making, goal‐directed behaviour and planning (Box 3).

Box 3 **Role of the prefrontal cortex in top‐down mediation of a stress response**

**The medial prefrontal cortex (mPFC) is a diverse region that mediates cognitive control**

The mPFC is often associated with executive functions such as decision‐making, rule encoding and goal‐directed behaviour. To translate these specific types of behaviour into neural mechanisms, it is important to better understand the underlying circuitry responsible for proper execution of behavioural output, especially as different types of behaviour often correspond to distinct functional groups of neurones. However, in general, neural ensembles in the mPFC appear to represent time‐sensitive rules and contingencies that confer a bias to guide downstream targets into an appropriate behavioural response.^204^

The mPFC can be organised into increasingly narrow functional units, based on their location, cell type or projection target. Topological distinctions can be made between prelimbic, infralimbic and anterior cingulate cortices, or even more generalised terms such as the ventromedial and dorsomedial prefrontal cortex.^205^ Occasionally, behaviour can be localised to one of these areas, although evidence for sparse and scattered neural ensembles encompassing much larger cortical areas continues to emerge as new techniques allow for increasingly selective observation methods.^206^ Previous work indicates that both the prelimbic and infralimbic cortex are important for anticipatory behaviour.^207^, ^208^ Moreover, different prefrontal interneurone types have been shown to shape decision‐making in various behavioural assays.^209^, ^210^ Finally, prefrontal projection neurones can have distinct roles based on their target region.^211^, ^212^, ^213^ In general, it appears that, although the mPFC exhibits a number of generalised core processes, such as having its neural ensembles exert top‐down influence on lower brain regions, the part that is activated depends on the type of behaviour.

**A change in environment that necessitates a response activates the mPFC**

A core function of the mPFC is the representation and imposition of behavioural strategy. For instance, neural ensembles in the mPFC can influence motor output by controlling neural activity in motor cortex areas or inhibiting inappropriate or impulsive responses.^208^, ^214^ However, when a change in the environment requires a different response than the one presented by the mPFC, new neural ensembles will be activated almost instantly.^215^ This is shown in various behavioural paradigms, indicating that the core function of the mPFC can be extended to include cognitive flexibility.

In the context of this review, recent literature provides some insight into possible interplay between the mPFC and a repeated stress source. It appears that the prefrontal cortex undergoes structural and functional modifications in response to a chronic stressor. Changes include synaptic remodelling and modulation of projecting neurones to subcortical areas. These adaptations appear to vary across mPFC subregions, where distinct projection targets and downstream networks from both prelimbic and infralimbic can be identified.^109^, ^110^, ^117^, ^123^, ^216^, ^217^

These structural and functional changes in response to a stressor could be an exponent of normal mPFC function. However, the manifestation of a stressor is not always the same, which complicates matters. A stress response could be elicited as a result of an emotional or internal stressor just as well as an environmental stressor. Although the mPFC is also involved in emotional flexibility, the pathways involved are dissimilar to those implicated in the cognitive domain. Moreover, the distinction between acute stress and chronic stress is an important one because the latter often involves larger systemic changes, which may overrule or partially sideline the mPFC, as exhibited in studies showing reduced mPFC function after chronic stress.^123^, ^218^, ^219^ In summary, incorporating a top‐down influence of the mPFC on the body's response to stress will require an integrated view of circuits underlying both emotional and cognitive flexibility.

### 4.4 Stress coping circuitry

Using optogenetics, a network responsible for coping was identified that originates from the prelimbic mPFC and innervates with glutamatergic efferents the anteroventral bed nucleus stria terminalis (avBNST)node.^117^ From there, a GABAergic network is activated that inhibits stress‐induced HPA axis activity.^118^, ^119^, ^120^ Another branch runs to the ventrolateral periaqueductal grey to execute the passive coping style during exposure to the inescapable tail suspension stressor.^120^ Previously, Keay and Bandler had shown that besides alignment of passive coping with ventrolateral central grey, the dorsolateral column mediates active coping with escapable stressors.^121^ Projections from infralimbic origin innervate the locus coeruleus and NTS, and also intercalate amygdala neurones that exert GABA‐ergic control over the central amygdala.^122^, ^123^ Prelimbic‐ and infralimbic PFC regions are important for fear expression and extinction.^122^

In very recent studies,^124^ lentiviral expression of mCherry conjugated to synaptophysin was used to identify monosynaptic projections from the infralimbic PFC. This analysis of structural connectivity was combined with optogenetic activation of infralimbic glutamatergic neuronal efferents and quantification of target neurone activation by c‐fos related antigen immunostaining. Such an approach revealed functional connectivity of the infralimbic PFC in particular with neurones of the medial dorsal thalamus, posterior hypothalamus and parts of the central/basolateral amygdala.^124^ Interestingly, some regions with extensive structural connectivity, such as BNST, showed only limited activation, but this discrepancy may be clarified when the database is extended with additional infralimbic ensembles and variation in environmental conditions. The infralimbic PFC communicates with nucleus accumbens and hippocampus, and projections exist from these regions back to the mPFC.^125^, ^126^

Acute stress promotes mPFC function; the rise in corticosterone enhances excitatory outflow as a result of GR‐mediated endocannabinoid blockade of GABA‐ergic interneurones.^127^ There is accumulating evidence showing that chronic stress or exogenous corticosterone infusion can compromise cognitive flexibility by atrophy of mPFC plasticity and connectivity.^128^ Exposure to a chronic variable stress paradigm down‐regulates GR expression in GABA‐ergic interneurones, thereby alleviating suppression of the inhibitory tone of these neurones on the excitatory outflux of the PFC pyramidal neurones. In a series of elegant experiments, McKlveen et al^129^ concluded causality between this “loss of a GR‐mediated brake on interneurone activity” and consequent increased synaptic inhibition of the PFC excitatory outflow. During chronic stress, the GR‐dependent hypo‐activity and atrophy of the mPFC eventually may lead to a disinhibited HPA axis and increased emotional behaviour, a condition that had been achieved previously by infralimbic GR knockdown.^130^

### 4.5 Priming of coping circuits

In a series of intriguing experiments over several decades, Maier and Watkins^110^ introduced the concept of “immunisation” or “inoculation” of the brain's fear circuitry by a previous stressful/fearful experience. It is assumed that, as a consequence, an enduring change in coping with future stressors is imposed. For this purpose, the brain circuit would be primed for dealing with future events. The dorsal raphe 5‐HT neurones appeared to be involved along with the infralimbic PFC‐intercalate interneurone‐central amygdala circuit and its output stations in the central grey responsible for expression of the fear‐induced freezing response. As explained in earlier above, the experience of a salient stressor appears to mobilise infralimbic PFC‐driven circuits with the goal to control the situation.^110^

This mechanism in higher brain regions possibly occurred later in evolution because it appears to proceed beyond the classical physiological defence reactions. The argument, however, is based on the measurement of HPA axis response pattern, which did not differ between escapable and inescapable stressors. Clearly, more rigorous experiments would be needed, such as performing the “priming” experiments in this paradigm under ADX vs adrenomedullectomy conditions or in site‐specific GR vs MR knockout animals. After all, MR and GR are expressed in stress circuitry and the role of these receptors in glucocorticoid control of selection and memory storage of coping style has been clearly demonstrated. Accordingly, the concept of priming or immunisation has great relevance; it is the basis of the single prolonged stress procedure that is widely used to model PTSD‐like symptomatology.^131^, ^132^

That the brain genome is profoundly changed after a chronic stress experience has been clearly demonstrated by an additional challenge 24 hours later. This challenge can be an acute heterologous stressor or just simply a corticosterone injection. Although, at baseline, the controls and chronic stress condition did not differ much in their gene expression pattern, the challenge revealed remarkable changes in hippocampal physiology and genomic organisation.^133^, ^134^ The number of genes underlying synaptic plasticity, development and epigenetic processes was over‐represented and the change persisted for some patterns (eg, NF‐κB) for several weeks.^104^ These observations support the notion proposed by Bruce McEwen et al 6 that: “one adapts but cannot roll the clock back.”

The inoculation of a stress coping style which is expressed during adult life, recapitulates the striking observation that early experience leaves its lifelong mark on physiological and behavioural stress reactions.^135^, ^136^ A variety of early‐life procedures, such as prenatal stress,^137^ handling,^1^ maternal care,^138^ repeated maternal separations,^139^, ^140^ a single 24‐hour deprivation procedure^141^ or limited bedding and nesting material,^142^ are all capable of causing epigenetic changes in genes encoding key regulators of HPA axis activity that underlie different trajectories of stress circuit organisation. For example, in rats, peripubertal programming to an adult aggressive phenotype may occur by repeated exposure to fear and stress.^143^ Strikingly, if the adult rat programmed for aggression was treated with the GR antagonist RU486, aggressive behaviour was abolished. The new data on prevention and/or reversal of stress‐induced programming effects add to a growing body of evidence demonstrating that blockade of the GR can *reset* stress response patterns imposed by (early life) trauma and chronic stress.^144^, ^145^

Presently, there are two hypotheses to explain later life consequences of early experience. First, dysregulation and increased vulnerability can occur when later life experience does not match the early‐life conditions: the match‐mismatch hypothesis.^140^, ^146^, ^147^ Second, there are the double hit, three hit or cumulative stress hypotheses, where an additional genetic load predisposes for a negative outcome from adversity experienced during perinatal and peripubertal life, which becomes expressed upon experience of a heterotypic stressor. Such a situation is encountered, for example, in animals that are genetically selected for increased brain dopamine function during adult life or for an enhanced peripubertal stress reactivity.^136^, ^143^

## 5 GLUCOCORTICOID MODULATION OF FEAR‐MOTIVATED BEHAVIOUR

Reduced circulating cortisol, and increased expression of GR with its downstream targets such as SGK‐1 and GILZ,^148^, ^149^, ^150^ are consistent biomarkers of trauma‐related vulnerabilities. In the brain, glucocorticoids target the amygdala‐prefrontal fear circuit via GR.^122^, ^151^ For example, central amygdala GR activation increases the expression of CRH and promotes fear‐motivated behaviour.^152^, ^153^, ^154^ The action of glucocorticoids on the different phases of information processing can therefore be exploited for better understanding of the mechanistic underpinning of trauma‐related vulnerability, which may precipitate PTSD symptoms in some individuals (Box 4).

Animal studies have indeed provided evidence of how treatment of anxiety disorders could benefit from specific modulation of either retrieval, (re)consolidation or extinction of fearful memory. First, glucocorticoids, acting via GR, promote memory consolidation of emotionally arousing information.^155^ Hence, in pharmacological experiments, the GR‐dependent consolidation of memory was found to be enhanced by NE agonists in animals and humans, and blocked by NE antagonists.^156^, ^157^, ^158^ The GR antagonist mifepristone *attenuates (re)consolidation of a cue‐conditioned fear response*, and this effect also required NE stimulation and, thus, could be prevented by a beta‐blocker.^159^

Box 4 **The clinical practice of trauma‐related psychiatric disorders**

1 Post‐traumatic stress disorder (PTSD) is a psychiatric disorder that can arise in the aftermath of trauma. It is characterised by a variety of symptoms, including intrusive memories, avoidance of traumatic reminders, negative alterations in cognitions and mood, and hyperarousal.^220^ Although diagnosis is possible as early as 1 month after trauma exposure, this interval is typically much longer as a result of avoidance and clinical delay. Once diagnosed, the first‐line treatment is trauma‐focused psychotherapy and this is effective in most cases; pharmacotherapy can also be indicated.^221^

Because PTSD is the only psychiatric disorder that must be preceded by a traumatic event to diagnose, clinical research on the impact of traumatic stress on health has focused on PTSD. This has resulted in numerous studies in PTSD patients that evaluate hypothalamic‐pituitary‐adrenal (HPA) axis and sympathetic nervous system functioning. Their collective aim has been to improve knowledge on the pathophysiology of PTSD to inform new diagnostic tools and improve treatment options. Unfortunately, meta‐analyses and reviews of these studies have yielded mixed results. The only consistent conclusion that could be drawn was that a substantial subgroup of PTSD patients exhibit an enhanced glucocorticoid feedback in the HPA axis. Accordingly, lower cortisol levels and increased glucocorticoid receptor (GR) expression were frequently reported.^20^, ^22^ A prospective study in deployed military personnel showed that trauma exposure was associated with an increase in functional GR‐1_F_ methylation and mental health problems but not PTSD.^150^ However, the relationship between trauma exposure and GR‐1_F_ methylation could not be replicated in a large cohort of early‐traumatised patients diagnosed with schizophrenia or bipolar disorder, or in siblings of these patients.^222^ Moreover, this epigenetic signature did not translate into an altered cortisol response to stress.

**The spectrum of psychiatric disorders impacted by trauma is much broader**

The abundance of opposing and/or inconclusive results is a widely acknowledged caveat in PTSD research. This is the reason why only selective serotonin reuptake inhibitors and a limited number of other pharmacological treatments are labelled as evidence‐based treatment options in the clinical guidelines for PTSD treatment. From a clinical perspective, this is not surprising because of the diverse characteristics of patients seen in clinical practice. These include early‐traumatised patients who had the bad luck of growing up in an unsafe and/or neglectful environment, patients working in high‐risk professions such as policemen or military personnel, patients who were involved in accidents, and the large and culturally diverse group of refugees. These patients have a lot in common in that they suffer from psychiatric problems with a large impact on daily functioning. They vary, however, in timing of trauma exposure during the lifespan, chronicity of trauma, coping styles and social support. They also differ in genetic and other vulnerability factors and, last but not least, in the presentation and treatment response. Moreover, studies conducted in patients diagnosed with personality disorders, depression,^223^ psychotic^224^ and bipolar disorders^225^ show that the spectrum of psychiatric disorders, which are impacted in onset and progression by trauma exposure, is much broader than formally acknowledged as trauma‐related.^226^

**GR and downstream genes are promising biomarkers for vulnerability and treatment**

Clinicians, and patients that do not respond to evidence‐based pharmacotherapy, are awaiting new treatment approaches, and glucocorticoid treatment paradigms are encouraging.^175^ To facilitate progress, it would be preferable to focus on the treatment of trauma‐related symptoms and refrain from the current diagnostic classification systems. In addition, since larger cohorts are needed, international collaborations will be essential for progress. For professionals at risk of developing trauma‐related disorders, insight into vulnerability factors is needed to selectively deploy preventive interventions. The expression of GR and possibly its downstream targets seem to be promising biomarkers to assess vulnerability and treatment in a subgroup of PTSD patients.^13^, ^20^, ^148^

Second, by the 1960s, glucocorticoids had already been shown to facilitate the extinction of fear‐motivated active and passive avoidance, and this action occurred independent of its suppression of HPA axis activity.^160^ In 2006, Cai et al,^161^ using the “freezing” response as a criterion for fear,^162^, ^163^ recapitulated these early studies and clearly demonstrated that glucocorticoids, acting via GR, could *facilitate extinction if given right after a single‐trial context‐dependent memory reactivation*. It was realised that the steroid does not merely act by erasing the fearful experience but, instead, activates GRs to promote the formation of a new memory of a situation that is now appraised as safe, or of no more relevance, or even rewarding.

Third, almost 40 years ago, we discovered a highly specific role of corticosterone in the so‐called forced‐extinction paradigm. In the one‐step‐through passive avoidance test, the retention of an acquired inhibitory avoidance response was not retained if the animals underwent ADX at 3 hours after the initial experience of the electric shock. Avoidance was re‐instated if, 1 hour prior to ADX, the animals were substituted with a low dose of corticosterone (30 μg s.c or 50 ng i.c.v. or even less in the hippocampus). Aldosterone, deoxycorticosterone, progesterone and dexamethasone were not active, although pretreatment could interfere with the corticosterone action. In retrospect, the experimental conditions illustrate *the role of activated MRs in appraisal processes*,* decision‐making and choice of behavioural coping strategy*, as outlined in the previous section.^164^

Finally, *retrieval of fearful experiences could also be a target* because it can be blocked by antagonising MR function^165^, ^166^ or activating GRs in an arousing context.^167^ Mutant mice engineered with forebrain MR overexpression and simultaneous global GR underexpression show the opposite phenotype: perseveration in searching for an escape route in the maze quadrant that previously harbored the platform. In the inhibitory avoidance test, the latency to re‐enter the dark compartment where the animal had been shocked was increased. Retention of shock avoidance remained high for at least 1 week and there was no sign of extinction with excess MR.^45^ Anti‐mineralocorticoids were anxiolytic in the elevated plus maze test.^168^ However, somewhat at variance with other data, virally mediated overexpression of MR in the basolateral amygdala was also found to be anxiolytic.^169^

To date, there are no clinical studies based on MR blockade as a means to interfere with retrieval. The first randomised controlled trial using GR activation to disturb retrieval (and facilitate extinction) showed a positive effect of cortisol treatment on reducing the re‐experience of PTSD‐like symptomatology and nightmares.^170^ A second study conducted in a larger cohort with patients receiving medication failed to replicate this outcome.^171^ Surís et al^172^ showed, in a double‐blind placebo‐controlled study, that glucocorticoid treatment after reactivation of memory reduced PTSD symptoms experienced 1 week later. Yehuda et al^173^ found that augmentation with cortisol around prolonged re‐exposure to trauma significantly reduced PTSD symptoms. Studies using high doses of glucocorticoids in emergency room settings showed a beneficial effect in preventing PTSD.^174^, ^175^ Cortisol also was effective in treatment of phobias in exposure‐based therapy.^176^ Why some of these treatment schedules were effective, even beyond the context of the fearful experience might be explained by the remarkable capacity of glucocorticoid receptor modulation *to reset* the stress system.^145^

## 6 PERSPECTIVES

One challenge for future research is to explore the mechanism of MR:GR‐mediated actions from the selection of an appropriate coping style all the way to memory storage of the experience to enable handling of similar threats in the future (Figure 1). In this respect, cortisol, MR/GR expression and their downstream targets in the brain qualify as trauma‐related vulnerability factors. Further knowledge of the mechanism of action would therefore help in the design of more specific GR (and MR) modulators with less side effects as a potential treatment strategy of dysfunctioning higher brain circuitry. This is of relevance for various mental disorders, including PTSD, in which early adversity, emotional neglect and trauma are recognised as vulnerability factors. The outcome of studies is encouraging when extinction of fear‐motivated behaviour is promoted. However, the translation of this knowledge to clinical practice is slow (Box 4).

Another great challenge is the understanding of prefrontal‐limbic midbrain circuit connectivity which exerts a top‐down influence on coping with stress. Optogenetics has identified specific downstream pathways from the mPFC via thalamic‐BNST‐amygdala switchpoints that regulate the physiologic stress reaction, behavioural coping and adaptation. Glucocorticoids feed back and modulate the plasticity and connectivity of this circuitry via MR:GR to enable management of the different phases of information processing. As indicated in Box 3, we are only beginning to understand how plasticity of mPFC neuronal ensembles is translated to cognitive flexibility underlying resilience.

The HPA axis and glucocorticoids have a key role in coping with stress. Moreover, the action of the renin‐angiotensin‐aldosterone system in stress adaptation is also becoming better understood, particularly in view of the highly specific NTS‐parabrachial‐BNST projections (Box 2). The brain demands approximately 30% of the body's energy resources.^177^ The fat‐brain axis has been identified as an important regulator of energy allocation to the brain, in addition to it being capable of modulating the physiological stress reaction, with a key role for the GR^12^ (Box 1). Interestingly, data mining revealed a remarkable abundance of receptors for metabolic signals (eg, leptin, insulin‐like growth factor‐1, ghrelin and insulin) linked to the fear circuitry, which may explain how metabolic status can regulate emotional state.^178^, ^179^ How, within the brain, energy is redistributed during large‐scale circuit dynamics over time^180^ is still poorly understood. In this respect, the action of glucocorticoids on mitochondrial function^84^ is opening novel insights into stress‐coping and adaptation.

## CONFLICT OF INTERESTS

ERdK owns stock of Corcept Therapeutics and is scientific advisor of the DynaCorts group.
